# The Obligations of Knowledge

**Published:** 1888-04-14

**Authors:** 


### The Hospital

A Weekly Institutional Journal of

Science, Medicine, Nursing, and Philanthropy.

*For Hospitals, Asylums, Medical Practitioners, Students, Nurses, Families, Parishes, Congregations, and the Charitable Public.*

Vol. IV. No. 81.] [April 14, 1888.

### The Obligations of Knowledge.

It seems certain that among the leading races of mankind there has been a gradual growth and strengthening of the moral faculties, of which the present result is that man is now more positively and uniformly moral than he has been at any previous period. All classes of writers who desire to gain the ear of thinking readers, clearly recognise and approve the general principles of scientific morality—such as justice, truthfulness, and the obligation of acting fairly, or doing to other people as they wish other people to do to them. It is one of the marked characteristics of modern agnostics and religious sceptics that they put forward the doctrine of Altruism, or self-denial, for the sake of the general good, as a principal claim to consideration. In novels of any pretence to literary merit there is always the same principle implied throughout, that the man or woman of genuine breeding is bound to regard the feelings, the wishes, the necessities, and even the prejudices of others with intelligent fairness; and in cases where it is possible to render a service, to render it spontaneously and naturally, as a part of ordinary conduct. So that we have among all men and women of culture a consensus of opinion and statement in favour of offering to other people every possible kind of service they may need at our hands, or that we may have it in our power to give.

If this be so, the doctrine of development is much strengthened on what has often been considered its weakest side. For the flower of the race to have attained such a position is a striking advance on "the good old rule, the simple plan, that he may take who has the power, and he may keep who can." It has the great and unspeakable advantage of being a point of agreement and unanimity among all kinds of men who desire to be virtuous and honourable. It is a position which has only been reached by centuries of metaphysics and practical experience. To certain persons it is as elementary as the letters of the alphabet; but as an article of belief for the variously-cultured few, its uniform adoption is comparatively recent; whilst as a principle of daily conduct for the many, it is still recognised very partially, and in numerous instances not at all.

We have affirmed that the cultured few, of all religious faiths and of none, whether sceptics, agnostics, positivisits, or pure theists, are entirely at one in the conviction that man owes duty to his fellow-man; that the measure of that duty is the degree of his power and opportunity for service; that he who fails to recognise and to render the fullest measure of good will and good deed to his fellows, not only stands condemned by the elementary canons of a reasonable religion, but must be regarded as a churl and an uncultured person by all men of light and leading of every school of thought.

Speaking of knowledge in the broadest sense, there is perhaps no obligation more weighty or more imperative than that of enlightening and informing the minds of mankind. All men of noble feeling and generous inspirations have felt a consuming zeal, an irresistible necessity laid upon them to make known to their fellows any moral principles, any scientific facts of which, by labour, by thought, or by intuition they may have become possessed. Names crowd upon us as the mind goes back over the fields of history, sacred and profane: a Moses, a Socrates, an Aristotle, a Spinoza, a Kant, a Hume, a Hobbes, a Hamilton, a Darwin, a Huxley. The history of the Christian Church furnishes records of hundreds—nay, of thousands—who, at the risk and loss of life, proclaimed to all who would heed, new moral principles, new standards of duty—principles and standards which, often opposed by the powerful and the wealthy, yet took root in the minds of the cultured and thinking few, and by their means were later adopted as the ideals, if not the daily rules, of the many.

The selfish and the vulgar desire knowledge for pride or for profit. Knowing more than their fellows, they can hold their heads higher and despise the rabble. Or, acquiring information of a special kind, they are able to deal it out with a niggard hand, always insisting upon a strict proportion between the amount of knowledge imparted and the weight and mint value of the standard coin to be received. Not thus does knowledge spread; not thus is science advanced; not thus is the human mind emancipated and made master of its environments; not thus is the faith of the people in its leaders and teachers strengthened; not thus shall the final triumph of intellect and virtue be achieved. The greatest book, which Carlyle tells us is not a book but a literature, warns us that "the people perish for lack of knowledge." What kind of knowledge is it the lack of which in these days of crowded cities causes men to perish? Is it only moral and religious knowledge? It is the want of the merest elements of sanitary and medical science which kills our children with diphtheria, our adults with typhoid fever, and our robustest intellectual workers with brain and nerve diseases.

Returning now to our starting-point, that all cultured men, of every faith and of none, recognise the duty, the imperative and finally-settled obligation, of rendering to all other men the fullest service of every kind which may be possible to them, we have to ask: How does the medical profession stand in this particular? The first thing to ascertain is the intellectual position of the practitioners of medicine in this regard. Is it abreast of the best culture of the time? As far as actual practice is concerned there is no doubt that medical men are more altruistic than any other class—at least, in certain departments of daily duty. But with regard to the undeniable and admitted obligation of imparting knowledge, where do they stand? We believe they stand in the forefront. It is true that within the last few months certain reactionary tendencies have been displayed; and even by a few in high quarters there has been evidenced a desire to claim anew for medicine the occult and esoteric character which was its degradation in superstitious and unscientific times. But the desire was limited and partial; the appearance of reaction was the result of timidity and misunderstanding. The really cultured, scientific, and right-minded leaders of the profession gave it no countenance; and now it is probable that some of the more worthy of the reactionaries are ashamed of their unworthy suspicions, and of the foolish and ungenerous position into which, for want of a rigorous logic, they allowed themselves to drift.

It is the clear and unchallengeable duty of the scientific medicine of the times, not only to give the public of its best in the way of treatment, but also in the way of instruction. The world has an easily-proved right to a degree of knowledge sufficient for self-defence. Taking the present position of vaccination as an illustration, the non-medical public ought to be so well informed of the scientific and experimental value of the successful operation that there should be no possibility of vaccination going largely out of use. In the same way the world ought to be well-informed enough to discriminate between the really capable and honest practitioner on the one hand and the showy and incompetent pretender on the other. In such a degree of knowledge there is the surest guarantee against incompetence and pretence.

To this position there can be no possible objection either in morals, in logic, or in expediency. The only plea which can stand for a moment is the plea that as the doctor has paid for his knowledge, so he is entitled to be paid in turn by those to whom he imparts it. That we readily grant; but just as the generous earth returns a hundred grains of wheat for every single seed that is sown, so will the physician, who is a man at the same time, not measure his services by the guineas he receives, but solely by the necessities of the world and his power to minister to them. The physician, we hold, is not a trader, who gives a measured amount of skill and knowledge in return for a measured sum in the current coin of the realm. Like the clergyman, he must be paid; but like the clergyman his ruling principle must always be "to do all the good he can to as many as he can." In so enlightening the world as to enlarge and strengthen its capacity for self-defence, not only is medicine acting in the noblest spirit of a true and victorious science, but it is taking the wisest of all courses to ensure its own safety, permanence, and progressive advancement. Let it never be forgotten that, "teaching, we learn," and "giving, we receive." "He that buildeth the gates of the city defendeth his own house."

